# Association between comorbidities and major adverse cardiovascular events in patients undergoing immune checkpoint blockade

**DOI:** 10.1186/s12885-026-16410-7

**Published:** 2026-06-27

**Authors:** Eveline Daetwyler, Annaïse Jauch, Núria Aeschlimann, Nikki Rommers, Benjamin Kasenda, Alfred Zippelius, Gabriela M. Kuster

**Affiliations:** 1 Division of Medical Oncology, Cantonal Hospital St. Gallen, Rorschacherstrasse 95, St. Gallen, CH-9007 Switzerland; 2 Division of Medical Oncology, University and University Hospital Basel, Basel, Switzerland; 3 Immunodeficiency Laboratory, Department of Biomedicine, University of Basel, University Hospital of Basel, Basel, Switzerland; 4 Department of Clinical Research, University of Basel, University Hospital Basel, Basel, Switzerland; 5 Department of Biomedicine, University of Basel, Basel, Switzerland; 6 Division of Cardiology, University Heart Center, University Hospital Basel, Petersgraben 4, Basel, CH-4031 Switzerland

**Keywords:** Immune-checkpoint inhibitor(s) (ICI[s]), Cardiovascular disease(s) (CVD[s]), Major adverse cardiovascular event(s) (MACE), Immune-related adverse events (irAEs), Long-term survival, Cancer

## Abstract

**Background:**

Cancer patients have an increased risk for cardiovascular diseases (CVDs). Conflicting information exists regarding whether cancer treatment with immune checkpoint inhibitors (ICIs) itself increases the risk for CVDs. We therefore assessed the incidence of major adverse cardiovascular events (MACE) in cancer patients treated with versus without ICIs.

**Methods:**

One hundred fifty-eight and 163 cancer patients, treated with and without ICIs respectively, were compared. The primary endpoint was the incidence of MACE, defined as acute coronary syndrome, ischemic stroke, transient ischemic attack, new or worsening coronary artery disease or peripheral arterial occlusive disease, or cardiovascular death. We used Cox proportional hazard models and competing risk models (death), adjusted for age, sex and comorbidities (Charlson Comorbidity index, CCI) to investigate the cause-specific association between ICI-treatment and MACE.

**Results:**

Over a median follow-up of 30.6 months, 37 MACE occurred. The overall incidence of MACE was 14.0% in patients with and 9.3% in patients without ICI-treatment. In the multivariate analysis CCI was significantly associated with MACE (HR 1.19, 95% CI [1.03, 1.37]), whereas no association was found for ICI-treatment (HR 1.12, 95% CI [0.56, 2.22]). Competing risk analysis showed no treatment associations. CCI was associated with non-cardiovascular mortality.

**Conclusions:**

We did not observe statistical evidence of an association between ICI treatment and MACE. There is evidence that a higher comorbidity burden is associated with MACE. Comorbidities are clearly associated with non-cardiovascular mortality in cancer patients. Further prospective studies are required to investigate whether treating comorbidities and optimizing cardiovascular risk factors result in improved outcomes among cancer patients.

**Graphical Abstract:**

**Figure:**
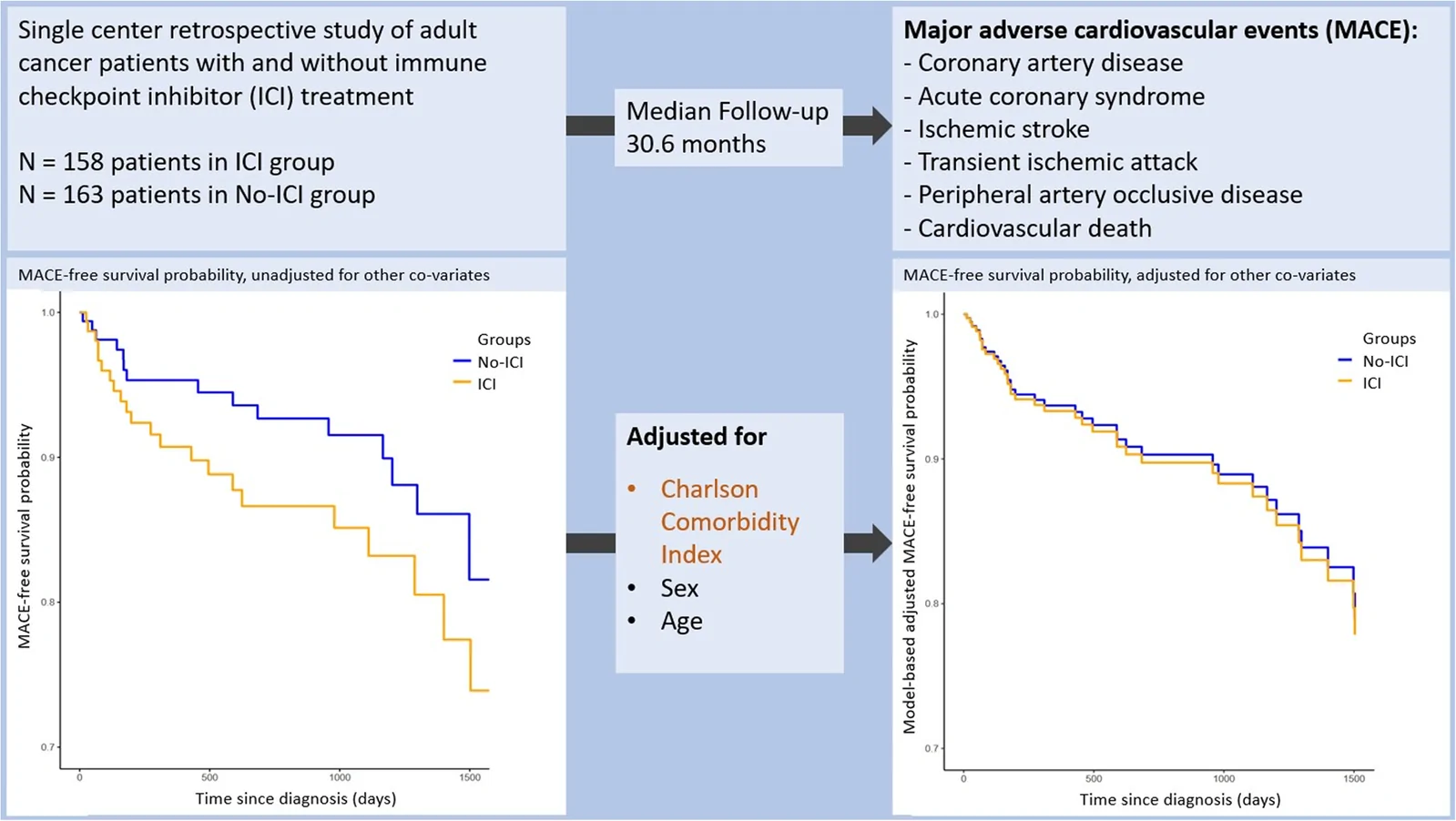

**Supplementary Information:**

The online version contains supplementary material available at https://doi.org/10.1186/s12885-026-16410-7.

## Background

Cardiovascular (CV) diseases (CVDs) and cancer are the leading causes of morbidity and mortality worldwide [1]. Given the substantial overlap in risk factors between cancer and CVDs [2, 3], assessing CVDs in cancer patients is essential, particularly in the light of improved life expectancy due to major advances in tumor treatment [4] and the potential long-term CV toxicities of cancer therapies. Similar to the general population, CVDs occurring in cancer patients cover a broad spectrum, among which atherosclerotic diseases such as acute and chronic coronary artery disease, ischemic stroke, transient ischemic attack and peripheral arterial occlusive disease have a high prevalence [3, 5, 6].

Shared modifiable risk factors for CVDs and cancer include smoking, alcohol, obesity, dietary factors, physical inactivity, hyperlipidemia, diabetes mellitus and high blood pressure [3, 6–9]. Non-modifiable risk factors, such as age, gender and previous history of CVDs, further increase the risk [3, 6–9]. These shared risk factors can induce chronic inflammation, oxidative stress, hormonal and metabolic imbalance including insulin resistance and dysregulated angiogenesis [6].

Most of these factors, individually, are important comorbidities that require attention in the treatment of cancer patients too. To quantify the burden of comorbid conditions and predict their mortality risk, the Charlson Comorbidity Index (CCI) was designed as a scoring system [10]. It assigns weighted scores to various pre-existing conditions, including cancer, certain CVDs and CV risk factors [10]. It has also been validated in lung cancer patients to provide a risk assessment for outcome and mortality [11–14].

Besides these risk factors, the anti-cancer treatment itself can contribute to the risk of developing CVDs. The adverse effects of radiotherapy and chemotherapy, such as for example anthracyclines, and recently also of targeted therapies on the CV system are mostly well established [15–17]. Over the last few years, the administration of immune-checkpoint inhibitors (ICIs) has gained importance in the treatment of cancers. Consequently, the use of ICIs is rapidly increasing, as around 40% of cancer patients in the United States were deemed eligible for ICI-treatment in 2019 [18]. Furthermore, ICI-treatment has substantially enhanced long-term survival, particularly in patients with metastatic melanoma [19]. Despite its effectiveness and success, it can lead to a broad range of immunological side effects, known as immune-related adverse events (irAEs) [20–22]. While these irAEs have been well described [20–22], it is still unclear whether ICIs increase the risk for CVDs due to a limited number of studies with discrepant results [23–29].

It has been suggested that ICI-treatment induces a low-grade inflammation of the arterial walls, resulting in progressive atherosclerosis, which leads to clinically evident CVDs [30–33]. Moreover, a few retrospective analyses report an increased risk of CVDs after ICI-treatment [23, 24, 26–28]. For example, Drobni et al. showed a 3-fold increased risk for developing an atherosclerosis-related CV event [24]. However, while these studies show an association of ICI-treatment and CV events, they often lack information about CV risk factors and underlying comorbidities. In contrast, studies in cohorts with only one cancer type show no influence of ICI-treatment on CVDs [25, 29].

Therefore, comprehensive evaluation of the impact of ICI-related CV effects across different risk factors and comorbidities is needed. To address this knowledge gap, we designed this observational study using routinely collected data. The primary objectives were to quantify the incidence of major adverse cardiovascular events (MACE) in cancer patients treated with systemic anti-cancer treatment and to investigate whether the administration of ICIs is associated with the occurrence of MACE, taking comorbidities into account. Moreover, potential risk factors for MACE were analyzed.

## Methods

### Study design

We conducted a retrospective, single-center cohort study using routine data of patients with a solid cancer diagnosis and who started their first systemic anti-cancer treatment at the University Hospital Basel, Switzerland, between 01/2018 and 12/2020. To identify patients, we reviewed chemotherapy protocols used in this period. We included all patients treated with ICIs in the first-line treatment (between 2018 and 2020) and who had provided a signed general research consent. To obtain an equal number of patients in the control group, we screened every third patient from all patients with systemic anti-cancer treatment without ICIs in the chemotherapy protocols. Exclusion criteria encompassed diagnoses other than solid cancers (e.g. hematological diseases), the presence of multiple cancers, initiation of anti-systemic cancer treatment before 2018, unknown treatment (e.g. study medication blinded), infeasible commencement of treatment due to the general condition and refusal to provide informed research consent (Supplemental Fig. 1). The ICI-group was defined as receiving ICI-treatment in the first-line treatment, irrespective of other concomitant treatments or following treatment lines. Patients who received ICI-treatment only in later treatment lines were excluded from the analysis. Patients in the No-ICI group did not receive any ICI-treatment during the course of the disease. Follow-up data of all patients were collected until July 2023. The study was approved by the Ethics Committee of Northwestern and Central Switzerland (2023–00832).

### Data capture

Standard demographic data, outcome parameters, comorbidities, CV risk factors and cardiovascular history were extracted from hospital records and entered into a REDCap database [34]. The following CV risk factors were included: diabetes mellitus, hypertension, hyperlipidemia, obesity/overweight, smoking (current/past), regular alcohol intake (defined as more than 3 drinks per week [one drink contains 14 g of alcohol] [35] or entry in medical records as a moderate/heavy drinker or alcohol use disorder), positive family history, history of CVDs, radiation of the heart region, renal insufficiency and sleep apnea. Diabetes mellitus, hypertension and hyperlipidemia were reported if they were listed in the patient files or if associated medications (antihypertensive, lipid-lowering, antidiabetic medications) were documented. Obesity was defined as a body mass index (BMI) of over 30 kg/m^2^ and overweight as a BMI between 25–30 kg/m^2^. CV history was defined as a history of coronary artery disease or acute coronary syndrome, peripheral arterial occlusive disease, ischemic stroke and transient ischemic attack. Cancer type, disease stage and systemic oncologic treatment were the cancer-specific covariates. Additionally, data on irAEs were collected. The following laboratory parameters were collected and deemed appropriate for analysis: CRP, leucocytes, neutrophiles, Troponin T, HbA1c, and lipid values (total cholesterol, LDL, HDL, triglycerides, lipoprotein(a)).

### Ascertainment of outcome

The primary objective was comparing the incidence of MACE in solid cancer patients with ICI-treatment to those with systemic anti-cancer treatment but without ICI-treatment. We considered the first event for each patient. This endpoint included a composite of acute coronary syndrome, newly diagnosed or worsening coronary artery disease or peripheral arterial occlusive disease, ischemic stroke, transient ischemic attack, and cardiovascular death. Acute coronary syndrome included ST segment- and non-ST segment-elevation myocardial infarction, and unstable angina as defined by the European Society of Cardiology guidelines [36]. We used the date of diagnosis as the starting point (time zero) for the time calculations, and patients without an event were censored at the date of the last contact. The diagnosis date was selected as the baseline (time zero) because it was consistently available for all patients and provided a standard clinical reference point for follow-up from the onset of the cancer disease course. This choice ensured that “time zero” was defined in a consistent manner for all patients treated with and without ICIs. Additionally, we aimed to identify risk factors that increase vulnerability to MACE.

### Statistics

All analyses were performed in R version 4.3.1 [37], with the alpha-level set at 0.05 [38]. Given the explorative nature of this study, we did not correct *p*-values for multiple testing. We describe the baseline patient characteristics stratified by treatment group (ICI group versus No-ICI group). We also describe treatment characteristics, CV risk factors, and blood biomarkers at the start of therapy by treatment group. Additionally, we report the incidence of all MACE, as well as the number of events per patient. We calculated “person years” by multiplying the overall number of patients by the time each patient spent in the study.

We fitted different models assessing risk for CVDs. For the primary outcome, the cause-specific hazard for MACE, we fitted a Cox proportional hazard model with treatment as the effect of interest and time to MACE as the outcome. Patients who have passed away before the occurrence of MACE or did not experience either MACE or death were censored at the date of death or at the date of last follow-up. Only the first event per patient was considered. Based on literature search and expert input, the model was adjusted for age, sex, and the CCI [39–42]. The proportional hazards assumption was assessed using scaled Schoenfeld residuals. No relevant violation of the proportional hazards assumption was detected for the primary Cox model. Kaplan–Meier curves were generated to visualize the MACE-free survival probability by group, both adjusted and unadjusted for the covariates in the model. With regards to the risk factors, we fitted Cox proportional hazard models to assess the individual associations with MACE, irrespective of treatment with or without ICI. These models were also adjusted for age, sex, and the CCI. For illustrative purposes we also fitted the cause-specific model (non-cardiovascular death) with the same adjustments.

As a secondary analysis, acknowledging the competing event of non-cardiovascular death, we fitted a Fine-Gray model with treatment as the effect of interest, MACE as the event of interest, and death due to other causes (non-cardiovascular death) as the competing event. The model was adjusted for the same set of pre-specified covariates.

As a supplementary analysis, we evaluated the association between irAEs and MACE in the ICI group using a Cox proportional hazard model, adjusted as for the primary analysis. The association was visualized with a Kaplan–Meier curve. In an additional supplementary analysis, we repeated the primary analysis in the lung cancer patients’ subgroup.

## Results

### Baseline characteristics

We included 158 cancer patients who were treated with ICIs (ICI group) at the University Hospital Basel between 2018 and 2020. To obtain an equal number of patients without ICI-treatment, we screened 564 patient records and finally included 163 patients (control group/No-ICI group) (Supplemental Fig. 1). Baseline and disease characteristics are summarized in Table 1. Overall, the two cohorts were similar with respect to age, sex, BMI, comorbidities, and CV risk factors. However, there were baseline differences. Patients in the ICI group presented more frequently with advanced/metastatic diseases, differed with respect to cancer type, showed higher mortality during follow-up, and had more often a history of past smoking.

**Table 1:**
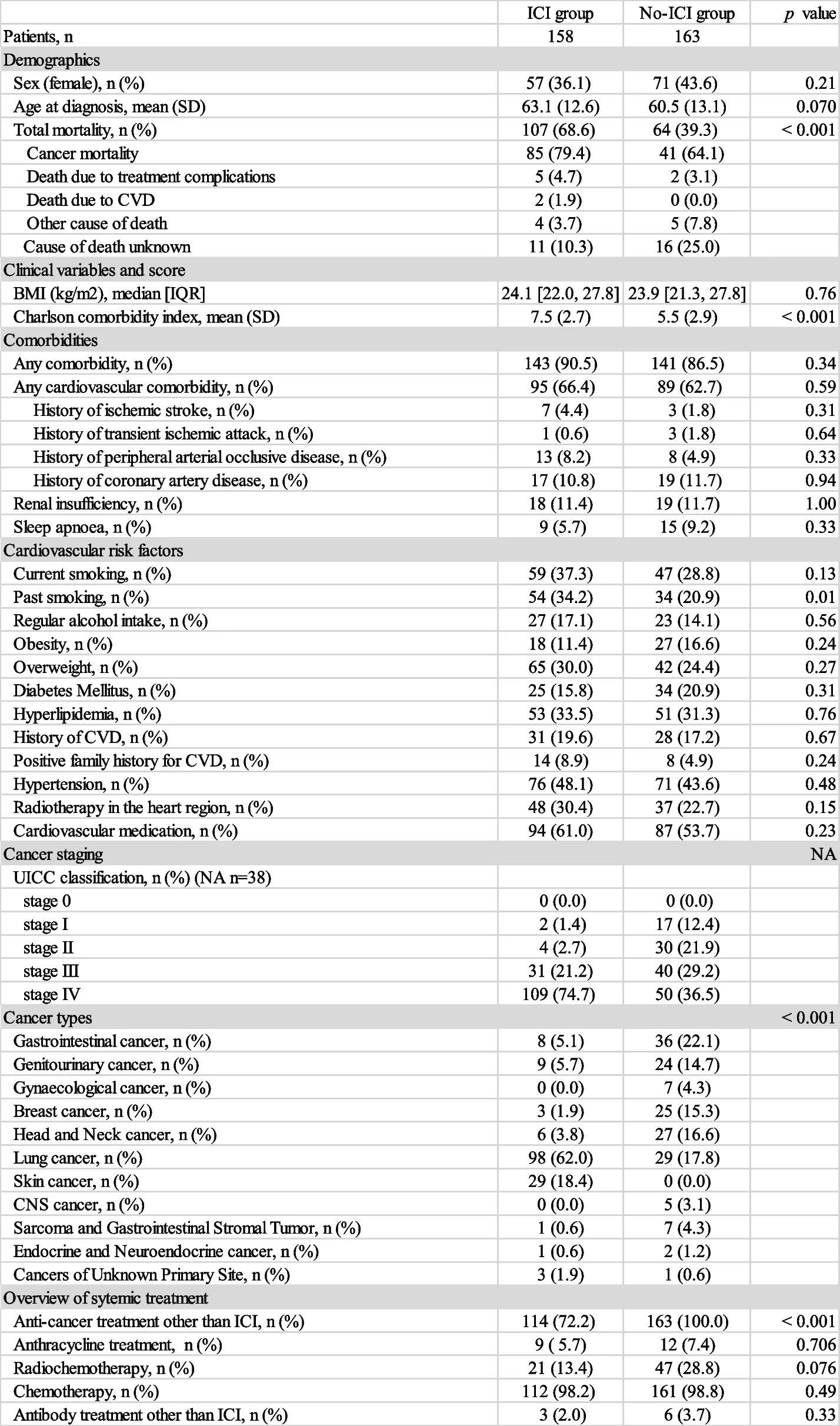
Baseline characteristics of patients treated with ICIs (ICI group) and without ICIs (No-ICI group)

Remark: History of CVDs includes history of ischemic stroke, transient ischemic attack, peripheral arterial occlusive disease, acute coronary syndrome, coronary artery disease

*Abbreviations*: *BMI* body mass index, *CVD* cardiovascular disease, *ICI(s)* immune checkpoint-inhibitor(s), *n* number, *NA* not assessed/not available, *UICC* Union for International Cancer Control

Lung cancer and skin cancer were the most common types of cancer in the ICI group; gastrointestinal cancer, lung cancer and head and neck cancer in the No-ICI group. The mortality rate was higher in the ICI group than in the No-ICI group, consistent with the increased number of patients with advanced cancer stage in the ICI group. The predominant cause of mortality was the underlying cancer (Table 1). CV medications were taken by 61% of patients in the ICI group, compared with 53.7% in the No-ICI group. No notable differences were observed between the two groups regarding the various types of CV medication (Supplemental Table 1).

### Major adverse cardiovascular events in cancer patients

A total of 37 first MACE were recorded during the total follow-up time of 746 person-years. Two CV deaths were observed in the ICI group; one CV death without a prior non-fatal MACE and one CV death after a prior non-fatal MACE. No CV deaths occurred in the No-ICI group. The median follow-up time per patient across all groups was 30.6 months. Specifically, in the ICI group, the median follow-up time was 25.0 months, while in the No-ICI group, it was 33.6 months. The incidence of MACE was 4.96 events per 100 person years. In general, 14.0% of patients who underwent ICI-treatment experienced a MACE, while the rate was 9.3% for those without ICI-treatment. The median time to event was 235.5 [92.2, 651.0] days in the ICI group and 455 [155.5, 1061.5] days in the No-ICI group, which was statistically not significant. Most of the events were the first occurrence of CVD. An overview of the specifications of the first event for each patient is presented in Table 2, stratified by treatment group.

**Table 2:**
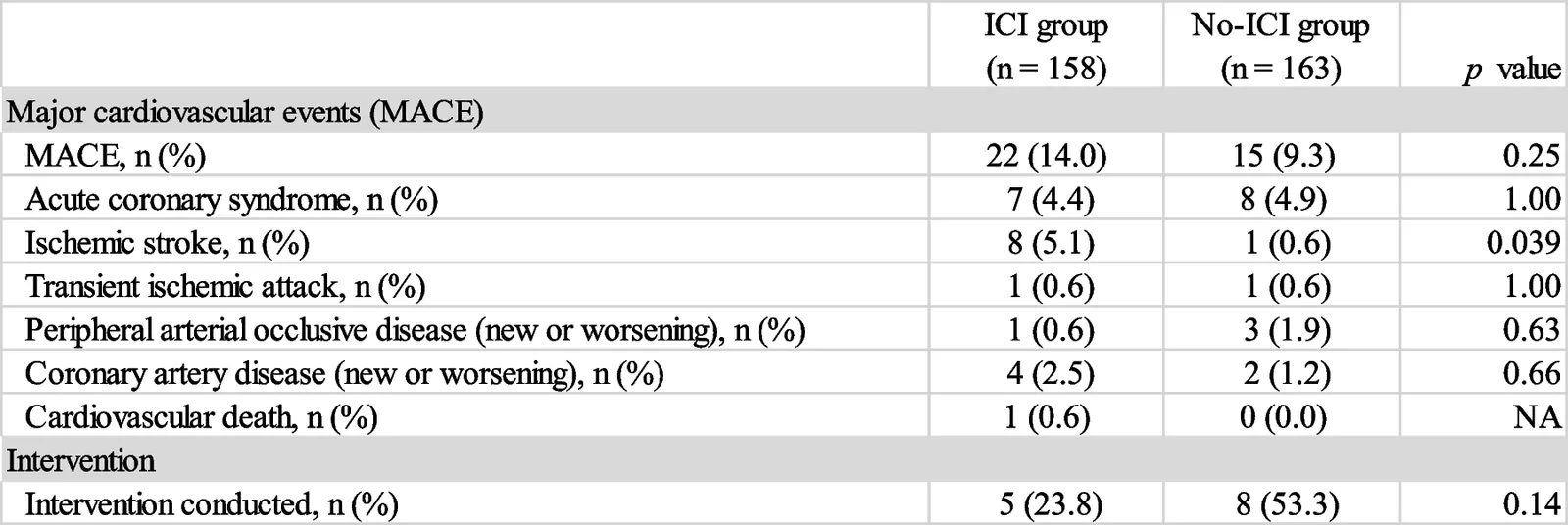
Overview of characteristics of MACE in patients treated with ICIs (ICI group) and without ICIs (No-ICI group)

Intervention comprised coronary re-vascularisation, cerebral lysis therapy, carotid endarterectomy and percutaneous transluminal angioplasty.

*Abbreviations*: *ICI(s)* immune checkpoint-inhibitor(s), *MACE* major adverse cardiovascular events, *n* number, *NA* not assessed

### Risk factors for major adverse cardiovascular events in cancer patients

The primary analysis showed that comorbidities, calculated by CCI, increased the risk for MACE (Table 3). With an increase in CCI of one point, the hazard increased by 19%. However, the data provided no evidence that there was an association between the risk for MACE and ICI-treatment (Table 3). When focusing on lung cancer patients only, ICI-treatment was also not associated with an increased risk for developing MACE (Supplemental Table 4). In the secondary analysis, accounting for the competing risk of death, CCI showed no significant association with the subdistribution hazard of MACE (Table 4). However, CCI was strongly associated with a higher risk of non-cardiovascular mortality (Table 5).

**Table 3:**
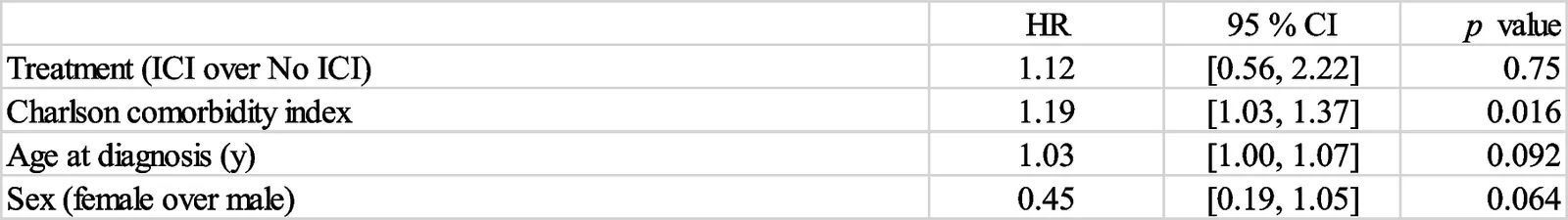
Hazard ratios from the proportional hazard model comparing the cause-specific hazard for MACE by treatment group (ICI versus No-ICI). The results for each covariate represent the effect after adjusting for all other variables in the model

*Abbreviations*: *CI* confidence interval, *ICI(s)* immune checkpoint-inhibitor(s), *MACE* major adverse cardiovascular events, *HR* hazard ratio, *y* year

**Table 4:**
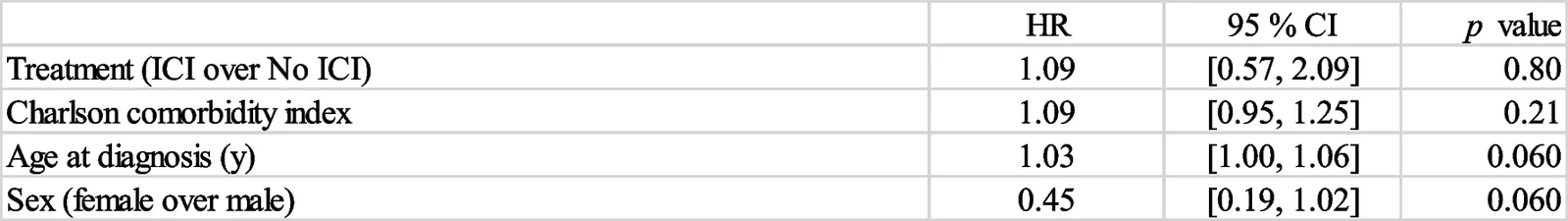
Subdistribution hazard ratios from the model comparing the subdistribution hazard for MACE by treatment group, including other-cause death as a competing event. The results for each covariate represent the effect after adjusting for all other variables in the model

*Abbreviations*: *CI* confidence interval, *ICI(s)* immune checkpoint-inhibitor(s), *MACE* major adverse cardiovascular events, *HR* hazard ratio, *y* year

**Table 5:**
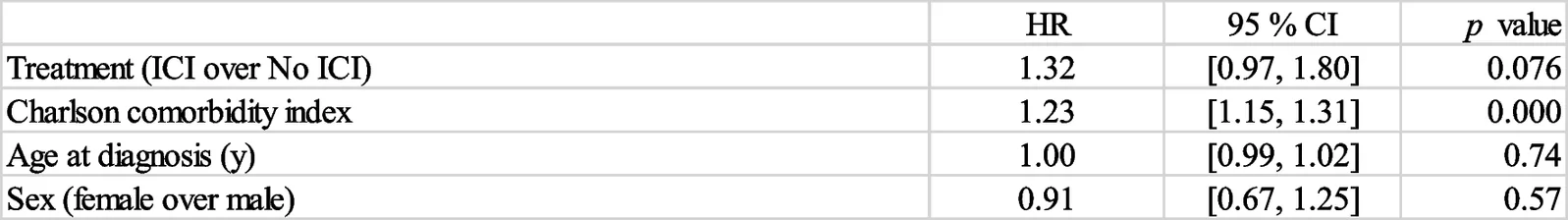
Hazard ratios from the model comparing the cause-specific hazard for non-cardiovascular death by treatment group. The results for each covariate represent the effect after adjusting for all other variables in the model

*Abbreviations*: *CI* confidence interval, *ICI(s)* immune checkpoint-inhibitor(s), *HR* hazard ratio, *y* year

**Fig. 1:**
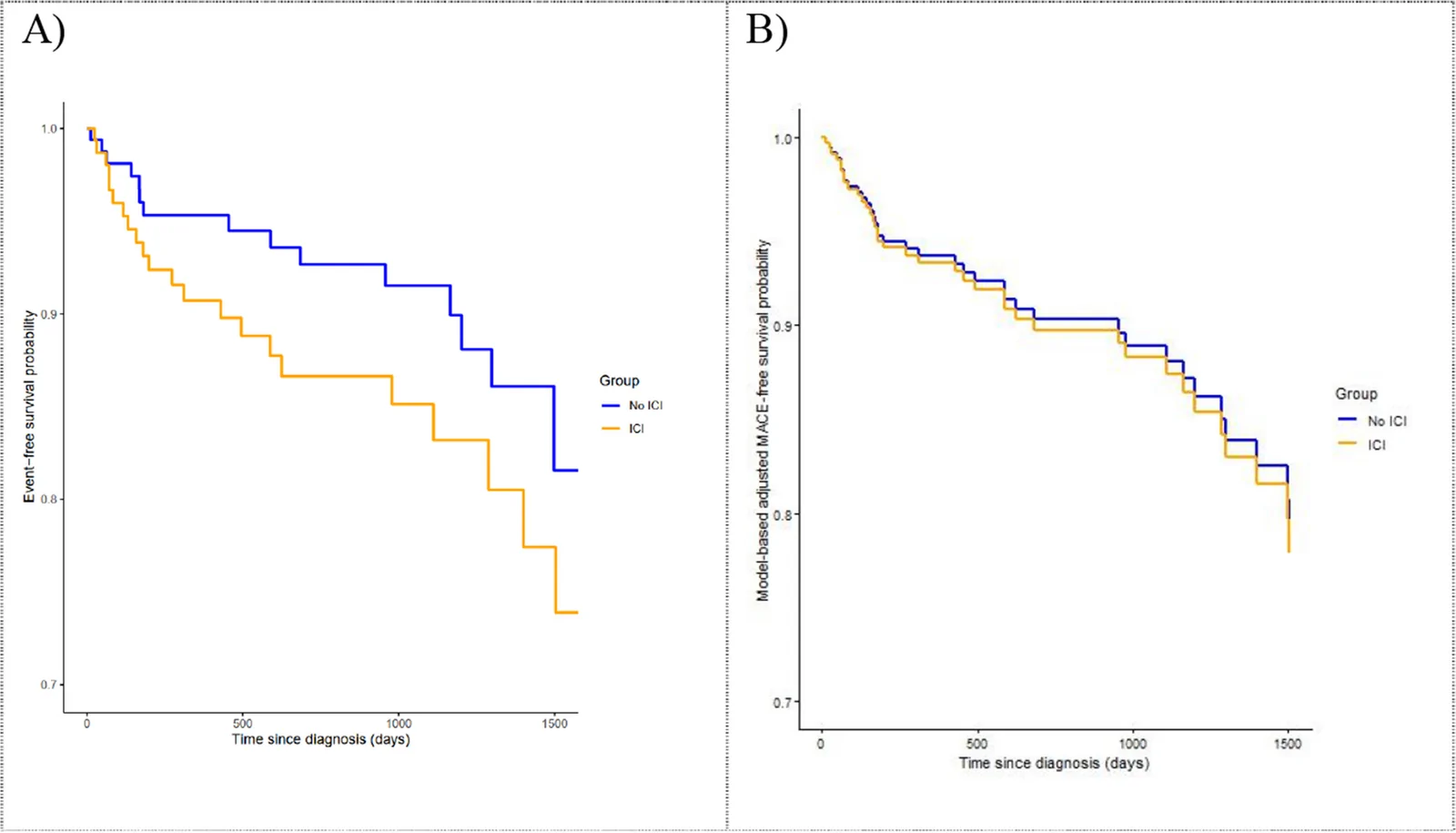
MACE in patients with ICI-treatment (ICI, in orange, *n* = 158 patients) and without ICI-treatment (No-ICI, in blue, *n* = 163 patients). **A** Unadjusted Kaplan–Meier curve. **B** Model-based adjusted MACE-free survival curve, standardized for age, sex, and CCI, using the Cox proportional hazards model. Because Fig. 1B is model-based, no directly interpretable number-at-risk table is shown for this panel. Abbreviations: CCI = Charlson comorbidity index, ICI(s) = immune checkpoint-inhibitor(s), MACE = major adverse cardiovascular events

When comparing MACE-free survival between patients receiving ICIs and those not receiving ICIs, unadjusted for other covariates, we observed lower MACE-free survival in the ICI group, although the difference did not reach statistical significance (Fig. 1A). Nevertheless, after adjusting for age, sex and CCI, the Kaplan–Meier curves overlapped (Fig. 1B). In the primary Cox model, ICI treatment was not statistically significantly associated with the cause-specific hazard of MACE (HR 1.12, 95% CI 0.56–2.22). The wide confidence interval indicates that both lower and higher hazards remain compatible with the data. The model-based adjusted curves were similar after adjustment for age, sex, and CCI, but residual confounding cannot be excluded.

Other risk factors for MACE were male sex, cardiovascular comorbidities, hyperlipidemia, obesity, and diabetes mellitus (Supplemental Table 2). The risk of MACE was over threefold greater when cardiovascular comorbidities or hyperlipidemia were present. Moreover, male sex consistently exhibited a higher risk for MACE. Additionally, there was no association between the occurrence of irAEs and MACE, when CCI, sex, and age were considered (Supplemental Fig. 2). Indeed, irAEs occurred in 45.8% of patients who experienced a MACE, compared with 46.5% of those who did not (Supplemental Table 3).

Furthermore, sensitivity analyses were performed to explore whether selected baseline imbalances influenced the estimated association between ICI treatment and MACE. Additional adjustment for smoking status did not materially change the treatment effect estimate (Supplemental Table 5). Similarly, after additional adjustment for metastatic disease status, ICI treatment did not show an association with MACE (HR 1.24, 95% CI 0.48–3.20, *p* = 0.66; Supplemental Table 6). Given the limited number of MACE, these sensitivity analyses were considered exploratory.

## Discussion

The successful treatment of cancer and the growing number of cancer survivors have shifted research focus towards investigating the long-term effects of cancer therapy and its associated risks. Particularly, the administration of highly effective ICI-treatment has improved cancer prognosis. However, various immunological side effects, referred to as irAEs, are associated with ICI-treatment [20–22]. In our study, we wanted to understand the implications of ICI-treatment on the development of CVDs. For this, we conducted a cohort analysis of MACE in patients treated with and without ICIs. Firstly, we found no evidence for a higher incidence of MACE in patients with ICI-treatment compared to patients without ICI-treatment. Secondly, there was some evidence that comorbidities, calculated by CCI, were associated with an increased risk for MACE, but this finding was not supported in our secondary competing risk analysis. However, the competing risk analysis showed that CCI was clearly associated with non-cardiovascular mortality. Lastly, we found that CV risk factors such as diabetes mellitus, obesity, hyperlipidemia, and male sex were associated with an increased risk for MACE.

In this retrospective cohort, we did not observe statistical evidence that ICI treatment was associated with a higher cause-specific hazard of MACE after adjustment for age, sex, and CCI. However, this finding should not be interpreted as evidence that ICI treatment does not pose a cardiovascular risk. The number of MACE was limited, the confidence interval for the treatment effect was wide, and baseline differences between treatment groups may not have been fully accounted for by the adjusted model. Nevertheless, this result adds to the ongoing debate about whether the use of ICI itself poses a risk for the development of CVDs. Our findings are supported by another single-center case–control study involving non-small cell lung cancer patients with a 6-month follow-up period. In this study, ICI-treatment was not independently linked to a heightened risk of MACE, defined as a composite of cardiovascular death, nonfatal myocardial infarction, nonfatal stroke, and hospitalization due to heart failure [29]. Similarly, Bar et al*.,* showed in patients with non-small cell lung cancer that there was no significant difference in venous and arterial vascular events when comparing patients treated with ICIs to those receiving chemotherapy [25]. In contrast, a comprehensive analysis of 59 oncological trials submitted to the US Food and Drug Administration, encompassing 21,664 patients, revealed a 35% increase in coronary ischemia over a 6-month follow-up period among patients receiving ICI-treatment compared with those undergoing chemotherapy [43]. Consistent with this result, Drobni et al. showed a 3-fold higher risk for MACE, including myocardial infarction, coronary revascularization, and ischemic stroke in cancer patients after starting ICI-treatment in a matched cohort study based on age, history of MACE, and cancer type, but not comorbidities [24]. Of note, in the subgroup of 40 melanoma patients who underwent assessment through computed tomography at three different time-points, ICI-treatment increased the rate of atherosclerotic plaque progression (from 2.1% per year before to 6.7% per year after ICI-treatment). Additionally, in a study by Laenens et al*.*, the incidence of a composite endpoint, consisting of acute coronary syndrome, heart failure, stroke, and transient ischemic attack, was higher (incidence 10.3%) in cancer patients treated with ICIs compared with those without ICIs, over a median follow-up period of 13 months [23]. It is noteworthy that in this study the effect was predominantly driven by the increased incidence of heart failure, which might be attributable to myocarditis or other myocardial processes and not atherosclerosis [44]. Moreover, there are varying results reported from imaging studies. For example, Calabretta et al*.* showed a significant increase in fluorodeoxyglucose (FDG) uptake across all aortic segments in 20 melanoma patients after ICI-treatment [45], whereas Poels et al., observed no absolute change in FDG-uptake in the large arteries in 10 melanoma patients treated with ICIs [46]. These conflicting outcomes could be attributed to variations in recording times and the limited sample size of patients.

Our study makes a substantial contribution to providing further clarification on this important question. To elucidate the variations in study outcomes, it is crucial to consider the influence of comorbidities. The absence of observable effects in studies concentrating on specific tumor types could suggest a more uniform distribution of comorbidities. This homogeneity may, to some extent, contribute to the comparable outcomes between patients receiving ICIs and those treated without ICIs. Furthermore, studies which showed that ICI-treatment was associated with MACE have not examined comorbidities or adjusted the results for this covariate. Without adjusting our results for comorbidities, there would be an inclination towards an elevated risk of MACE with ICI-treatment. However, after accounting for factors such as sex, age, and CCI, the disparity between the ICI group and the No-ICI group vanished.

Comorbidities, as quantified by the CCI, independently elevated the risk of MACE. This effect was not seen in the model with the competing risk analysis. Considering the patients enrolled in the study, there was a large overlap in the risk factors between the event of interest (MACE) and the competing event (non-cardiovascular death), as CCI demonstrated a strong association with the cause-specific hazard of both MACE and death. This dual influence likely diluted the effects of CCI on the subdistribution hazard of MACE in the competing risk model.

The CCI encompasses certain pre-existing CVDs like myocardial infarction, peripheral arterial occlusive disease, cerebrovascular diseases, along with CV risk factors such as age, diabetes mellitus, and impaired kidney function. This might explain the strong association between CCI and MACE. Further research is required to elucidate the different aspects contributing to the CCI in relation to MACE in cancer treatment. So far, the CCI has been validated with respect to overall survival in cancer patients [11–14] and also in patients with CVDs, such as acute coronary syndrome [47]. To our knowledge, our study is the first to show the applicability of this score in assessing the risk of MACE in cancer patients.

Additionally, our Cox proportional hazard models demonstrated a susceptibility to experiencing MACE among individuals with the following risk factors: pre-existing CV comorbidities, hyperlipidemia, diabetes mellitus, obesity, and male sex. These findings are consistent with previous studies, in which CV risk factors increased the vulnerability to developing MACE [23–25]. As these risk factors are strong drivers for the development of CVDs, it is important to consider them in the formulation of prevention strategies as recommended by CV guidelines [7, 48–52]. In the literature, sleep apnea [53] and renal insufficiency [54] are also recognized as comorbidities associated with an increased risk of developing CVDs. Nevertheless, our study did not uncover a statistically significant association, which is likely attributable to the relatively limited number of patients in each group.

Furthermore, we investigated irAEs and CV irAES separately as potential risk factors for MACE. There are indications that excessive stimulation of the immune system, similar to a flare-up in autoimmune diseases, may lead to the progression of subclinical atherosclerosis through inflammation [55–57]. In our study, there was neither an association between acute CV irAEs and occurrence of MACE, nor with irAEs affecting other organ systems during our follow-up of 2.3 years (median). This finding aligns with recent data where no correlation was observed between immune-related endocrinopathies and acute vascular events [25].

Lastly, the importance of comorbidities in cancer patients independent of ICI-treatment warrants further optimization of disease management, toxicity monitoring protocols, and evaluation of preventive treatment strategies [58, 59]. This discovery highlights the importance of adopting a collaborative healthcare approach for cancer patients, emphasizing interdisciplinary collaboration among various specialists, including cardiologists, oncologists, and physicians specializing in other internal medicine fields. In addition, our study further highlights the importance of considering comorbidities in future research on the CV risk of patients treated with ICIs.

### Limitations

This study was designed as a retrospective, single-centre analysis including all eligible patients available during the study period. The study design implies inherent methodological limitations that must be acknowledged. The retrospective nature of the study introduces the possibility that unmeasured confounding variables may have influenced the observed associations, and some patient data were absent as they were not recorded according to a predefined study setup. To mitigate model overfitting, variable selection was carefully restricted to clinically relevant parameters, including age, sex and comorbidities, measured with the CCI-score. While this approach ensured the stability of the model, it also resulted in a reduction in the capacity to explore additional variables that could potentially contribute to the observed associations. However, additional analysis investigating the associations between MACE and potential confounders, as well as their interaction with treatment, did not reveal additional confounders.

Another limitation relates to the heterogeneity of the cancers in the study population. The patient cohort comprised of individuals with different types of cancer and treatment regimens. Separate analyses were performed for the largest subgroup, lung cancer; nevertheless, the statistical power remained limited. Due to the small sample size and unequal group distribution, adjustments for treatment-related factors, particularly the use of anthracyclines, were not feasible. The limited overall number of patients similarly constrains the validity of subgroup comparisons and the identification of cancer type–specific effects on the development of CVDs.

Overall, the relatively small sample size and number of events limit the statistical power of our study, particularly for addressing specific questions such as cancer type specific effects on CVDs, and restrict the generalizability of the findings, which should therefore be interpreted as hypothesis-generating. Prospective, multicenter studies with standardized data collection and larger cohorts will be required to confirm these observations and clarify the mechanistic links between MACE and ICI-treatment.

## Conclusions

In this retrospective cohort, we did not observe statistical evidence of an association between ICI treatment and MACE after adjustment for age, sex, and CCI. The comorbidities, however, appear to be associated with the risk of developing MACE. CCI appears to be the most important prognostic factor, irrespective of the applied treatment. Therefore, an in-depth assessment of the comorbidities in cancer patients is essential. We strongly recommend that patients with an elevated CCI, pre-existing CVDs and CV risk factors undergo a thorough CV evaluation prior to starting ICI-treatment. These patients might also benefit from closer follow-up monitoring, referral to specialized cardio-oncologists and evaluation of comprehensive preventive strategies. This is particularly important as MACE are associated with a high morbidity and mortality [23, 44].

## Supplementary Information

Supplementary Material 1.

## Data Availability

The data that support the findings of this study are not openly available due to reasons of sensitivity and are available from the corresponding author upon reasonable request.

## Abbreviations

BMI: Body mass index
CCI: Charlson comorbidity index
CV: Cardiovascular
CVD(s): Cardiovascular disease(s)
ICI(s): Immune checkpoint inhibitor(s)
FDG: Fluorodeoxyglucose
irAE(s): Immune-related adverse event(s)
MACE: Major adverse cardiovascular event(s)
n: Number
NA: Not available/not assessed
