# Supplementary material for: Association between comorbidities and major adverse cardiovascular events in patients undergoing immune checkpoint blockade
Source: BMC Cancer. 2026 Jun 27;26:1094. doi: 10.1186/s12885-026-16410-7 (PMC13576238; doi:10.1186/s12885-026-16410-7)
Supplement: Supplementary file 1 [file 12885_2026_16410_MOESM1_ESM.docx]

**Supplemental Material**

**Association between comorbidities and major adverse cardiovascular events in patients undergoing immune checkpoint blockade**

Eveline Daetwyler, MD, ^1,2^, Annaïse Jauch, PhD, ^2,3^, Núria Aeschlimann, MD, ^2^ Nikki Rommers, PhD, ^4^, Benjamin Kasenda, PhD, ^2,4^, Alfred Zippelius, MD, ^2,5*^, Gabriela M. Kuster, MD, ^5,6*^

^1^ Division of Medical Oncology, Cantonal Hospital St. Gallen, St. Gallen, Switzerland

^2^ Division of Medical Oncology, University and University Hospital Basel, Basel, Switzerland

^3^ Immunodeficiency Laboratory, Department of Biomedicine, University of Basel, University Hospital of Basel, Basel, Switzerland.

^4^ Department of Clinical Research, University of Basel, University Hospital Basel, Basel, Switzerland

^5^ Department of Biomedicine, University of Basel, Basel, Switzerland

^6^ Division of Cardiology, University Hospital Basel, Basel, Switzerland

* shared last authorship

**Supplemental Table 1**: Baseline characteristics of patients treated with ICIs (ICI group) and without ICIs (No-ICI group)

Abbreviations: CR = complete response, ICI(s) = immune checkpoint-inhibitor(s), n = number, N/A = not applicable, PD = progressive disease, PR = partial response, SD = stabile disease

**Supplemental Table 2**: Hazard ratios from the models assessing the different risk factors, adjusted for age, sex and CCI

Abbreviations: CCI = Charlson comorbidity index, CVD = cardiovascular disease, HR = hazard ratio, hs = high sensitive

**Supplemental Table 3**: Grading of irAEs in patients with and without a MACE (only in patients treated with ICI)

Abbreviations: CV = cardiovascular, ir = immune-related, irAE = immune-related adverse events, MACE = major adverse cardiovascular events, n = number

**Supplemental Table 4**: Hazard ratios from the model comparing the hazard for CV events by treatment group only in lung cancer patients. The results for each covariate represent the effect after adjusting for all other variables in the model.

Abbreviations: CI = confidence interval, CV = cardiovascular, ICI(s) = immune checkpoint-inhibitor(s), MACE = major adverse cardiovascular events, HR = hazard ratio, y = year

**Supplemental Table 5:** Hazard ratios from the model comparing the hazard for CV events by adjusting for CCI, age, sex and past smoking.

Abbreviations: CV = cardiovascular, CI = confidence interval, ICI(s) = immune checkpoint-inhibitor(s), HR = hazard ratio, y = year

**Supplemental Table 6:** Competing-risk analysis using a Fine–Gray subdistribution hazards model for MACE with non-cardiovascular death as the competing event, adjusting for M-stage, Charlson comorbidity index, age, sex, and a treatment-by-M-stage interaction for each covariate representing the effect after adjusting for all other variables in the model.

Abbreviations: CI = confidence interval, ICI(s) = immune checkpoint-inhibitor(s), MACE = major adverse cardiovascular events, HR = hazard ratio, y = year

**Supplemental Figure 1**: Identification and screening of patients in the No-ICI group.

564 patients in the No-ICI group were screened. 401 patients were excluded due to following reasons: non solid cancer diagnosis, presence of multiple cancers, treatment start before 2018, unknown systemic treatment or start of the treatment not possible and refusal of the informed consent.

Abbreviations: n= number

**Supplemental Figure 2**: Model-based MACE in patients with irAEs (orange) and without any irAEs (blue) in the ICI group.

Kaplan Meier curves were used to show the MACE free survival probability for patients suffering irAEs (n=54 patients at 0 days) versus patients without any irAEs (n=104 patients), adjusted for age, sex and CCI.

Abbreviations: CCI = Charlson comorbidity index, ir = immune-related, irAE = immune-related adverse events,
MACE = major adverse cardiovascular events
